# Complete chloroplast genome of *Physalis chenopodifolia* Lam. (Solanaceae)

**DOI:** 10.1080/23802359.2019.1698364

**Published:** 2019-12-11

**Authors:** María del Pilar Zamora-Tavares, Isaac Sandoval-Padilla, Abigail Chávez Zendejas, Jessica Pérez-Alquicira, Ofelia Vargas-Ponce

**Affiliations:** a Departmento Botánica y Zoología, Instituto de Botánica, Centro Universitario de Ciencias Biológicas y Agropecuarias, Universidad de Guadalajara, Zapopan, Mexico;; b Maestría en Biosistemática y Manejo de Recursos Naturales y Agrícolas, Centro Universitario de Ciencias Biológicas y Agropecuarias, Universidad de Guadalajara, Zapopan, Mexico;; c Laboratorio Nacional de Identificación y Caracterización Vegetal (LaniVeg), Cátedras CONACyT, Universidad de Guadalajara, Zapopan, Mexico;; d Departamento de Botánica y Zoología, Centro Universitario de Ciencias Biológicas y Agropecuarias, Universidad de Guadalajara, Zapopan, Mexico

**Keywords:** Husk tomato, plastome, phylogeny, tomatillos, Solanaceae

## Abstract

*Physalis chenopodifolia* is a perennial wild tomatillo with traditional use in central Mexico because of its edible fruits. Due to their agronomic potential and nutraceutical properties, this species is a resource that can be a candidate to plant breeding programs to be included in the Mexican diet. Here, we report the complete chloroplast genome of *P. chenopodifolia*. Its full size is 156,888 bp, includes a large single-copy (LSC) region of 87,117 bp, a small single-copy (SSC) region of 18,451 bp, and two invert repeat (IR) regions of 25,660 bp each. The plastome contains 113 genes, 79 protein-coding genes, 4 rRNA genes and 30 tRNA genes. The phylogenetic hypothesis supports *P. chenopodifolia* as a member of *Physalis* genus. Although relationships within the genus have moderated bootstrap support, the utility of the complete plastome sequence to solve infrageneric phylogenetic relationships is confirmed.

*Physalis chenopodifolia* Lam. is a perennial herb endemic to Mexico. It grows in oak, pine, tropical deciduous forest, grasslands, and xerophilous scrubs from Chihuahua in the north, to Oaxaca in the south. Its fruits are orange berries with reddish tones, bittersweet in taste, and is eaten as season’s fruit by the people of Mazahua origin (Valdivia-Mares et al. 2016). The roots, stems, and fruiting calyx are used in traditional medicine (Santiaguillo Hernández and Blas 2009). Due to their phytochemical, nutraceutical properties, and agronomic potential, this species undergo plant breeding programs as an agro-alimentary resource (Valdivia-Mares et al. 2016; Salcedo-Pérez et al. 2018). This work characterizes the sequence of the complete chloroplast genome of *P. chenopodifolia* that will be useful for future genetic studies.

Fresh leaves of *P. chenopodifolia* were collected in Huejotzingo Puebla (19°04′17.6″N, 98°30′4.07″W). The cDNA was isolated according to Shi et al. (2012) and stored at the Laboratorio Nacional de Identificación and Characterización Vegetal (LaniVeg) at the Universidad de Guadalajara (Voucher: OVP539-05112011). A total of 615,321 single-end reads were generated in Ion Torrent PGM (Thermo Fisher Scientific, Carlsbad, CA) and *de novo* assembly with SPAdes 3.12.0 (Bankevich et al. 2012). The assembled genomic sequences were filtered with Bowtie2 2.3.5 (Langmead and Salzberg 2012). The annotation was performed with GeSeq (Tillich et al. 2017). The protein-coding genes and rRNA were confirmed with BLAT (Kent 2002) and the tRNAs with tRNAscan-SE 2.0.3 (Chan and Lowe 2019). Plastome circular representation (GenBank accession number MN508249) was generated with OGDraw 1.3.1 (Greiner et al. 2019).

The plastome size of *P. chenopodifolia* is 156,888 bp. The genome exhibits the typical quadripartite circular structure. It has a large single (LSC) region of 87,117 bp, a small single (SSC) region of 18,451 bp, and two inverted repeated (IR) regions of 25,660 bp. The nucleotide composition was 30.83% adenine, 19.08% cytosine, 18.44% guanine, and 31.65% thymine. General GC content was 37.52%, but IR had 43.06% each, the LSC and SSC showed 35.57% and 31.36%, respectively. The plastome included 113 genes, 79 protein-coding, 4 rRNA and 30 tRNA. The IR has 21 duplicate genes that corresponded to 4 rRNA, 8 tRNA, 7 protein-coding genes and 2 pseudogenes. Additionally, 16 genes comprised introns, 2 genes with 2 and 14 with 1.

To understand the phylogenetic position of *P. chenopodifolia* within Solanaceae, we used the plastome of *P. philadelphica* Lam. (Sandoval-Padilla et al. 2019) and 18 plastome sequences of Solanaceae species (downloaded from NCBI GenBank database). Additionally, we used *Ipomoea batatas* (Convolvulaceae) as an outgroup. The sequences were aligned using MAFFT v7.307 (Katoh and Standley 2013). Maximum Likelihood (ML) analysis was performed using RAxML (Stamatakis 2014) and 1000 bootstraps iterations with the evolutionary model GTR + I + G, according to the estimate of jModelTest 2.1.10 (Darriba et al. 2012). The phylogenetic hypothesis supports *P. chenopodifolia* as a member of *Physalis* genus (Figure 1). Although the relationships of the *Physalis* species exhibited moderate bootstrap support, the complete plastome sequence is useful to infer phylogenetic relationships within this genus.

**Figure 1.**
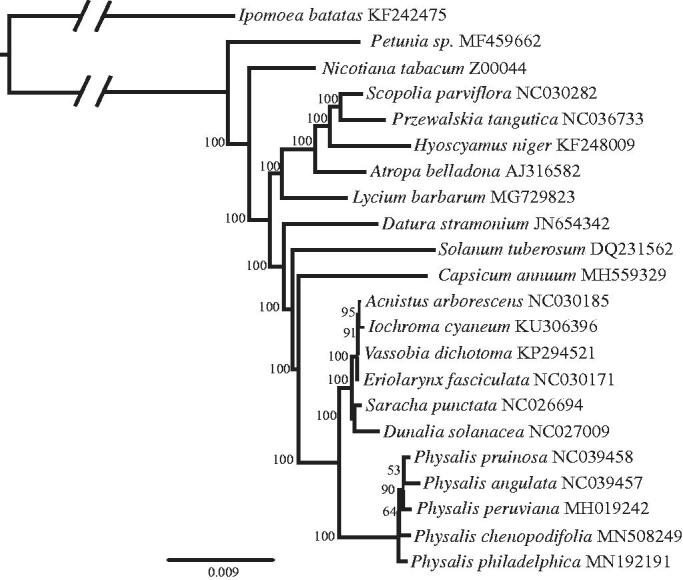
The maximum-likelihood (ML) tree of 21 Solanaceae species including *Physalis chenopodifolia*. Bootstrap value based on 1000 replicates are shown in the nodes. GenBank accession numbers are shown after the species name.
